# Zebrafish mutants reveal unexpected role of Lrp5 in osteoclast regulation

**DOI:** 10.3389/fendo.2022.985304

**Published:** 2022-09-02

**Authors:** Iryna Khrystoforova, Chen Shochat-Carvalho, Ram Harari, Katrin Henke, Katherine Woronowicz, Matthew P. Harris, David Karasik

**Affiliations:** ^1^ Azrieli Faculty of Medicine, Bar-Ilan University, Safed, Israel; ^2^ Department of Orthopedics, Emory University, Atlanta, GA, United States; ^3^ Department of Orthopaedics, Boston Children’s Hospital, Boston, MA, United States; ^4^ Department of Genetics, Harvard Medical School, Boston, MA, United States

**Keywords:** lrp5, zebrafish, bone, osteoclast, osteoporosis

## Abstract

Low-density Lipoprotein Receptor-related Protein 5 (*LRP5*) functions as a co-receptor for Wnt ligands, controlling expression of genes involved in osteogenesis. In humans, loss-of-function mutations in *LRP5* cause Osteoporosis-Pseudoglioma syndrome, a low bone mass disorder, while gain-of-function missense mutations have been observed in individuals with high bone mass. Zebrafish (*Danio rerio*) is a popular model for human disease research, as genetic determinants that control bone formation are generally conserved between zebrafish and mammals. We generated *lrp5-* knock-out zebrafish to study its role in skeletogenesis and homeostasis. Loss of *lrp5* in zebrafish leads to craniofacial deformities and low bone mineral density (total body and head) at adult ages. To understand the mechanism and consequences of the observed phenotypes, we performed transcriptome analysis of the cranium of adult *lrp5* mutants and siblings. Enrichment analysis revealed upregulation of *genes* significantly associated with hydrolase activity: *mmp9, mmp13a, acp5a*. *acp5a* encodes Tartrate-resistant acid phosphatase (TRAP) which is commonly used as an osteoclast marker, while Matrix metalloprotease 9, Mmp9, is known to be secreted by osteoclasts and stimulate bone resorption. These genes point to changes in osteoclast differentiation regulated by *lrp5*. To analyze these changes functionally, we assessed osteoclast dynamics in mutants and observed increased TRAP staining, significantly larger resorption areas, and developmental skeletal dysmorphologies in the mutant, suggesting higher resorptive activity in the absence of Lrp5 signaling. Our findings support a conserved role of Lrp5 in maintaining bone mineral density and revealed unexpected insights into the function of Lrp5 in bone homeostasis through moderation of osteoclast function.

## Introduction

Osteoporosis is a debilitating condition characterized by compromised bone architecture and decline of bone mineral density (BMD) associated with aging. Worldwide, osteoporosis affects millions of people and may result in bone fracture (1), causing heavy burden on the health, social and economic aspects of life (2, 3). Osteoporotic fractures require long-term immobilization and rehabilitation that consequently may contribute to loss of life (4). Over the last century, human lifespan has extended. A consequence of this extended lifespan is an enhanced emphasis on the skeletal system as a source of significant morbidity associated with aging (5). Continued turnover of bone extracellular matrix sustains skeletal integrity and maintains structure of bone as can be measured by mineral density of the bones (6). This turnover is controlled *via* a coupled interaction of bone-building osteoblasts and resorbing osteoclasts (7). The disparity of these integrated actions may result in low bone accrual and lead to BMD loss with age (8, 9).

Low-density Lipoprotein Receptor-related Protein 5 (LRP5), a co-receptor of the canonical Wnt-pathway, was identified as a locus associated with fracture risk and BMD by large-scale genome-wide association studies (GWAS) (10–13). Multiple studies have provided evidence that LRP5, is critical for bone development, growth, and maintenance (14–17). Together with Frizzled, LRP5 controls expression of genes involved in osteogenesis (18). These findings are supported by studies describing mutations in *LRP5* that underlie different pathological conditions and affect bone metabolism in humans (18). Loss-of-function mutations in LRP5 cause osteoporosis-pseudoglioma syndrome (OPPG), a low bone mass disorder, with severe low BMD and pathological vascularization of the retina (19), while point mutations having a gain-of-function affect have been observed in individuals with high bone mass (20). Mutations in *LRP5* are also known to be associated with cranial deformities, such as torus palatinus and thickening of frontal and mandible bones (Van Buchem disease) (21).

Animal models mirror the necessity of *lrp5* in postnatal bone metabolism and closely resemble human phenotypes. It has been shown that murine *Lrp5* knock-out models mimic the human OPPG phenotype with low BMD and appendicular limb deformities (16, 22). In murine *Lrp5* knock-outs the main cause of poor bone quality and low mineral deposition is mostly ascribed to reduced osteoblasts proliferation (14, 23). However, less is known about the effect of *Lrp5* knock-out on osteoclasts function. It was shown that high bone mineral density missense mutations in *Lrp5* reduce osteoclast differentiation and resorption activity in female mice (24). However, the systemic effect of *lrp5* deficiency on osteoclasts more broadly remains unknown.

The zebrafish has been a key vertebrate model to understand gene function in development and physiology (25). There are many advantages of zebrafish as an animal model: external development, high fecundity, optical clarity of embryos and larvae, short generation time, ease of genetic manipulations, availability of mutant and transgenic lines and, most importantly, high genetic similarity with humans (26, 27). Notably, zebrafish bones demonstrate similar cellular content and share molecular mechanisms with mammalian bones (28–30). As a result, zebrafish bones and tissues became a faithful model to study human bone disorders (28).

In the present study, we generated zebrafish *lrp5* knock-outs that allowed us to analyze bone phenotypes at various stages. Our model demonstrates delayed mineralization throughout the zebrafish skeleton at early stages with further deformation of the neuro- and viscerocranium in the adult craniofacial skeleton. We found that adult *lrp5* ^-/-^ fish had low BMD and displayed malformations of the craniofacial skeleton that may be a result of parasphenoid bone fractures. Surprisingly, transcriptome profiling of *lrp5* ^-/-^ cranial bones revealed the downregulation of genes in the bisphosphonate pathway together with mevalonate pathway genes, known as key pathways for controlling osteoclasts metabolism. Using the zebrafish model, we show that osteoclasts in *lrp5^-/-^* mutants are more active, resulting in increased bone resorption compared to *lrp5^+/+^*. Thus, our work uncovers an unexpected role of Lrp5 in suppressing osteoclast regulation in addition to its effect previously identified on bone mineral density. We suggest that the *lrp5* ^-/-^ zebrafish model can contribute to identifying treatments to ameliorate symptoms of low BMD and prolong the health of aging bones.

## Materials and methods

### Zebrafish husbandry

All experiments were conducted according to institutional animal care and use committee (IACUC*)* approval for Bar-Ilan University zebrafish facility (protocol #b13213_40). Zebrafish (*Danio rerio*) of AB strain were maintained at 28°C under 14:10 light : dark cycle.

### Generation of *lrp5^-/-^* line by CRISPR-Cas9

CRISPR-Cas9 was used to knock out *lrp5* in zebrafish. The plasmids pCS2-nCas9n (47929) and pT7-gRNA (46759) were purchased from Addgene (Cambridge, MA, USA). Two DNA oligomers 5`-TAGGGTCGCTCAGAGTCTGCAG-3` and 5`-AAACCTGCAGACTCTGAGCGAC-3` were annealed and ligated in to the pT7 gRNA plasmid after it was digested with BsmBI, BglII and SalI. gRNA was generated by *in vitro* transcription of the pT7-gRNA digested with BamHI, using T7 RNA polymerase (New England Biolabs, Ipswich, MA, USA) followed by purification using MicroSpin G-50 Columns (GEHealthcare, San Diego, CA, USA). For making Cas9 mRNA, the template DNA (pCS2-nCas9n) was linearized by NotI, purified using a QIAprep column, capped Cas9 mRNA was synthesized using mMESSAGE mMACHINE SP6 kit (Invitrogen) and purified using Micro Spin G-50 Columns. A mix of *lrp5* gRNA (300ng/μL) and Cas9 mRNA (300ng/μL) was injected directly into zebrafish one-cell-stage embryos, using a pneumatic Pico Pump (WPI, Worcester, MA, USA).

After 24 hours the CRISPR efficiency was evaluated by PstI-HF (New England Biolabs) as the restriction site of this enzyme was located at the CRISPR-Cas9 target site. Next, CRISPR injected embryos were raised, and DNA was extracted from their fin tail and analyzed by PstI-HF to select founders carrying mutations. Founder fish were outcrossed to WT and DNA was extracted from several embryos of each founder and sequenced to decipher the mutation transmitted to F1 offspring. Further, F1 carrying an 8bp deletion in exon 2, were outcrossed with WT to generate F2 which were again outcrossed to WT to generate F3 to reduce off target effect. The *lrp5* ^-/-^ and *lrp5* ^+/+^ siblings were obtained by inbreeding of F3 *lrp5* ^+/-^.

### Genotyping

For DNA extraction, embryo or adult fin tail were immersed in 100μL of 50mM NaOH heated to 95°C for 20 minutes and cooled down to 4°C. Afterwards, 1/10 volume of 1M Tris-HCL buffer at with pH8 was added (31). 5 μL of DNA was used for PCR with specific primers (**Table S1**) and PCR fragments were either cut by PstI-HF and run on a 2% agarose gel or sequenced (Hy-lab, Rehovot, Israel).

### Western blot

The total proteins from caudal fins of adult fish were extracted by incubation in RIPA lysis buffer (Sigma-Aldrich, Burlington, MA, USA) with protease cocktail (Sigma-Aldrich) and separated in sodium dodecyl sulphate–polyacrylamide gel electrophoresis (SDS-PAGE). The proteins were transferred to polyvinylidene fluoride (PVDF) membrane (Merck Millipore Ltd, Tullagreen, Ireland) and blocked by 4% skim milk in Tris-buffered saline with 0.1% Tween-20 (TBST) v/v. Next, membrane was immunolabeled with anti-*LRP5* rabbit monoclonal antibodies (1:1000, D80F2, Cell Signaling Technology, Danvers, MA, USA) or anti-α-Tubulin mouse monoclonal IgM antibodies (1:1200, TU-02, sc-8035, (Santa Cruz Biotechnology, Dallas, TX, USA). Secondary antibodies (1:1500, ab97240, Goat Anti-Mouse, and 1:1500, ab97051, Goat Anti-Rabbit, Abcam) were incubated for 1 hour at room temperature. Membranes were developed using chemiluminescent substrate (Advansta Inc., San Jose, CA, USA) and imaged *via* UVITEC Alliance Q9 Imager (Cleaver Scientific, Warwickshire, UK).

### Skeletal staining

#### Alizarin red alcian blue skeletal staining

To visualize bone and cartilage tissues at 7, 13 days post fertilization (dpf) and 1.5 month post-fertilization (mpf), *lrp5* ^-/-^ and *lrp5* ^+/+^ siblings were stained according to Walker and Kimmel protocol (32). In short, fish were euthanized and fixed in 4% paraformaldehyde (Sigma-Aldrich), for 2 h, washed with phosphate buffered saline (PBS), and dehydrated with 50% and 70% ethanol. Next, larvae were double stained with 0.5% alizarin red (Sigma-Aldrich) and 0.02% alcian blue (Sigma-Aldrich) or stained with alizarin red only, overnight, washed with distilled water and bleached with 3% H_2_O_2_ (Merck Millipore) and 2% potassium hydroxide (KOH, Sigma-Aldrich). Larvae were incubated at 37°C in 1% trypsin (Biological Industries, Beit HaEmek, Israel) in 2% borax (Sigma-Aldrich) solution with mild agitation for 45 min until 60% of the soft tissue was dissolved clear. Afterwards, larvae were serially washed with 20% glycerol mixed with 0.25% KOH, and 50% glycerol mixed with 0.25% KOH for 2 h. The larvae were photographed using Leica M165 FC microscope. Percentage of proximal notochord’s calcification (stained red) was measured using ImageJ software (NIH, Bethesda, MD, USA).

#### Von Kossa staining

Elasmoid scales were collected from 10 mpf old fish and washed 3x in PBST, then stained in 2.5% of silver nitrate (2.5% AgNO_3_ in dH_2_O, Sigma) solution for 30 minutes. Stained scales were washed in PBST and then placed for 5 min in 5% sodium thiosulfate solution and washed again before transferred into 80% glycerol (33). Scales viewed under an automated upright slide scanning microscope, Axio Scan.Z1 (Zeiss, Oberkochen, Germany). The images were captured with 40×/0.95 objective at z-planes of 0.5 μm. Analysis of the resorbed area was done in ImageJ Software with use of ZFBONE toolset (34). 12 fish per group and at least 10 scales per fish were analyzed.

#### Tartrate‐resistant acid phosphatase staining

To detect osteoclasts an acid phosphatase kit (387A; Sigma‐Aldrich) was used. Scales were collected and fixed in fixative solution at room temperature and then washed 3x in PBST. TRAP staining solution was prepared following kit protocol. Scales were stained for 3h at 37°C in 500µL of TRAP stain. Then scales were washed in PBST and re-fixed in 4% PFA at 4°C overnight. Prior to imaging, scales were washed in PBST and moved to 80% glycerol in PBST. The images were captured with 10×/0.95 objective at z-planes of 0.5 μm. Analysis of the osteoclast’s activity was done in ImageJ software with threshold application. 10 fish per group and at least 10 scales per fish were analyzed.

### X-ray micro-computerized tomography

At 3 and 6mpf zebrafish were euthanized and fixed in 3.7% formaldehyde (Sigma-Aldrich) in PBS overnight. Length, weight and sex were recorded, and fish were stored in 70% ethanol until scanning. A total of 55 fish were scanned (3mpf: *lrp5^-/-^* n=15, *lrp5^+/+^* n=15; 6mpf: *lrp5^-/-^* n=12, *lrp5^+/+^* n=13) using a 1172 SkyScan micro-computed tomography (micro-CT) scanner (Bruker, Kontich, Belgium). The whole body of fish was scanned at pixel size of 12 μm (scan settings 49 kV, 100 μA, filter Al 0.25mm). The scanned files were reconstructed with NRecon Software (Bruker). The BMD was measured from reconstructed files using CTAnalyzer Software (Bruker) calibrated to the phantoms with known mineral density (0.25 and 0.75 g.cm^−3^ hydroxyapatite, Bruker). The 3D tomography images of zebrafish whole body were generated using CTvox software (Bruker). The morphometrical measurements of nasofacial angle (NA) and parasphenoid (PD) distance between two limits were done on 3D reconstructed micro-CT scanned zebrafish using Meshy online tool from GitHub platform (https://0x00019913.github.io/meshy/). Prior to the measurements, the reconstructed zebrafish heads were saved in 3D models using CTAnalyzer Software (Bruker).

### RNA-sequencing and data analysis

Total RNA was extracted from 10mpf old zebrafish craniums (*lrp5^-/-^* n=8, *lrp5^+/+^* n=7). The tissue was lysed in Trizol (Sigma-Aldrich) and purified by Direct-Zol RNA kit (Zymo Research, Tustin, CA, USA). Integrity of the isolated RNA was tested using the Agilent TS HS RNA Kit and 4200 TapeStation at the Genome Technology Center at the Faculty of Medicine at Bar-Ilan University. 100 ng of total RNA was treated with the NEBNext poly (A) mRNA Magnetic Isolation Module (NEB, #E7490L). RNA-seq libraries were produced by using the NEBNext Ultra II RNA Library Prep Kit for Illumina NEB #E7770L. Quantification of the library was performed using dsDNA HS Assay Kit and QUBIT (Molecular Probes, Life Technologies Corporation, Gaithersburg, MD, USA) and qualification was done using the Agilent TS D1000 kit and 4200. 250nM of each library was pooled together and diluted to 4nM according to NextSeq manufacturer’s instructions. 1.6pM was loaded onto the Flow Cell with 1% PhiX library control. Libraries were sequenced by Illumina NextSeq 500 platform with single end reads of 75 cycles according to the manufacturer’s instructions.

### Differential expression analysis

We compared the gene expression between *lrp5^-/-^* and *lrp5^+/+^* control siblings by mRNA differential expression (DE) analysis. The sequenced reads of all samples were aligned to the zebrafish genome (GRCz11) using TopHat2 (35), and the number of reads mapped within genes was quantified by Quantify to annotation model (Partek E/M) (36) of the Partek Flow software, v 10.0. This procedure resulted in a sequencing depth of 30–35 million reads per sample, out of which 80–81% were uniquely mapped to the zebrafish genome (GRCz11). Then, low expressed transcripts (<100 reads in all samples combined) were filtered out and data was normalized to trimmed mean of M-values in the Partek Flow. One outlier in the *lrp5^-/-^* group was detected by principle component analysis and removed. Differentially expressed genes were determined using the GSA (gene specific analysis) program from the Partek Flow package. P-values were corrected for multiple testing using the Benjamini–Hochberg method, with genes with a false discovery rate (FDR) < 0.05 and absolute value fold change (FC) >2 classified as differentially expressed. List of differentially expressed zebrafish genes was converted to their human orthologs using DIOPT (37), keeping only human orthologs with a DIOPT score >6. In cases where there were multiple zebrafish orthologs for one human gene, the gene with the highest log fold change in expression was used. Gene enrichment analysis was done using the online tool ShinyGO (http://bioinformatics.sdstate.edu/go/) with WikiPathways database.

### Quantitative real-time PCR

For RNA extraction the tissue was lysed in Trizol (Sigma-Aldrich) and purified by Direct-Zol RNA kit (Zymo Research, Tustin, CA, USA). cDNA was synthesized from 1µg RNA by Takara PrimeScript kit (Takara, Mountain View, CA, USA). qPCR was performed with PowerUp SYBR Green Master Mix (Thermo Fisher Scientific, Waltham, MA, USA) using a ViiA™ 7 Dx qPCR Instrument (Life Technologies Corporation). All reactions were performed as technical triplicates, and non-template controls were added in each PCR run. The relative expression values of the genes were normalized to the housekeeping control (primers are listed in **Table S2**).

## Results

### Generation of Lrp5 loss of function mutant zebrafish

**Figure 1:**
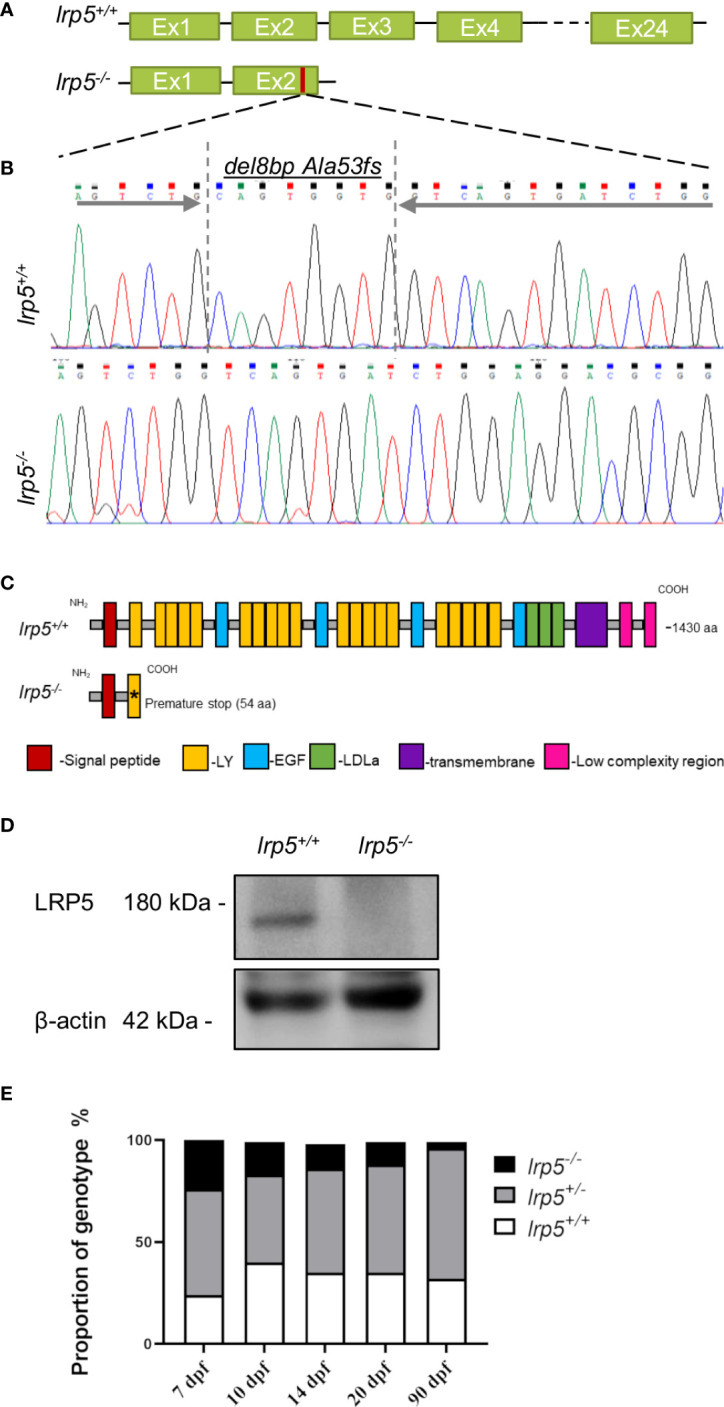
CRISPR-Cas9 induced *lrp5* zebrafish knockout line. **(A)** Schematic representation of the ZF *lrp5* gene structure with Sanger sequence chromatogram; **(B)** demonstrating the del8bp (Ala53fs) mutation, that results in a frameshift and a premature stops codon; **(C)** Schematic representation of the ZF LRP5 wild-type and mutated protein demonstrates the lack of functional domains in LRP5 of *lrp5^-/-^* (LY -low-density lipoprotein receptor; EGF – epidermal growth factor; LDLa- Low-density lipoprotein receptor domain class **(A, D)** Western-blot results demonstrated the absence of LRP5 protein in *lrp5^-/-^.* **(E)** Each column in the graph represents the proportion of genotype (%) per developmental stage. Only 3.7% of *lrp5^-/-^* in survive and reach adolescence using instead of expected 25%.

LRP5 protein and function is highly conserved between human and zebrafish (38). The zebrafish Lrp5 protein (NP_001170929.1) consists of 1430 amino acids residues. Using CRISPR-Cas9 mediated genome editing we induced an 8 base pair (bp) deletion in exon 2 of the *lrp5* gene (**Figures 1A, B**), which resulted in a frame shift at alanine 53 (Ala53fs), creating a premature stop codon after 54 amino acids. The truncation resulted in the absence of the functional protein domains, including the transmembrane region (**Figure 1C**). The LRP5 protein level in the *lrp5^-/-^* mutants was verified by western-blot, demonstrating a complete loss (**Figure 1D**). *lrp5^-/-^* zebrafish were further used to follow skeletogenesis from larval to adult stages to directly compare its role in regulating skeletal differentiation and development.

Very low numbers of homozygote fish were obtained through incrossing of carriers (**Figure 1E**) with only of 3.7% homozygotes surviving through adolescence (90 dpf); heterozygous fish did not display any reduction in survival rates. On closer inspection, we found that from 7 dpf the number of *lrp5^-/-^* fish decreased; daily manual water changes up to 1mpf increased survival of *lrp5^-/-^* mutants allowing for a broader analysis of the phenotype.

### *lrp5* is necessary for timing and extent of mineralization during development

**Figure 2:**
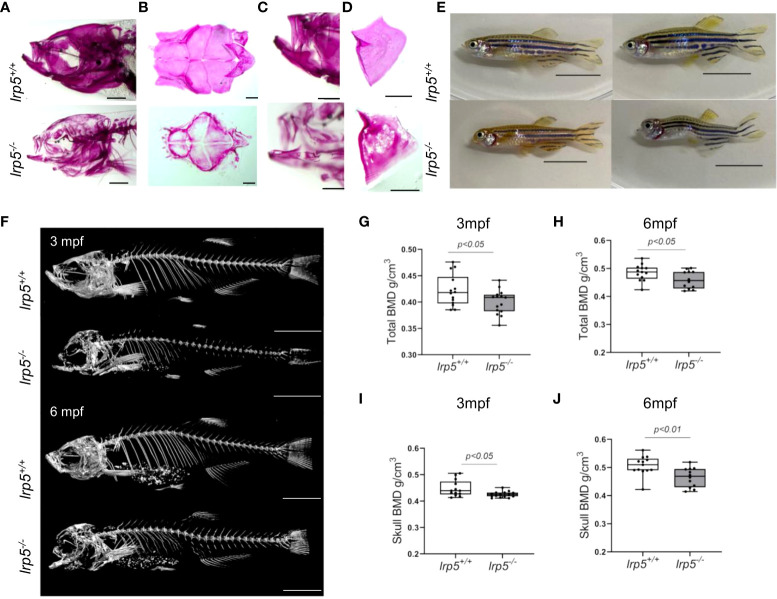
*lrp5* ^-/-^ adult fish display low bone mineral density. **(A)** Whole-mount alizarin red staining at 1.5 mpf display the distribution of mineralized matrix through the head bones. In the skull of *lrp5^-/-^* distribution of mineralized matrix throughout whole head bones differs from *lrp5^+/+^* Scale bar=1mm. **(B)** coronal view of the cranial vault. The alizarin red signal is distributed ubiquitously in *lrp5^+/+^* lower jaw **(C)** and opercle **(D)**, meanwhile in *lrp5^-/-^* fish the lower jaw demonstrated discontinues alizarin red signal. Scale bar=500µm. **(E)** Representative photos of *lrp5^+/+^* and *lrp5^-/-^* siblings at 4mpf; *lrp5^-/-^* present with deformed head and body curvatures. Scale bar=1cm. **(F)** 3D reconstruction of X-ray images of *lrp5^+/+^* and *lrp5^-/^* ^-^ skeleton at 3 and 6mpf. Scale bar= 1cm **(G, H)** Total BMD is decreased in adult *lrp5^-/-^* fish. **(I, J)** the skull BMD of *lrp5^-/-^* mutant fish is significantly lower both at 3 and 6mpf. Results in G-J are expressed as mean SD (3mpf n =13-15 fish, 6mpf n =12-13 fish, t-test).

As early as 7dpf several of the craniofacial skeleton elements start to mineralize, such as the notochord (**Figures S1A, D**, outlined in yellow) and can be easily observed by alizarin red staining for mineralized matrix. Using alizarin red staining alone (**Figures S1A, D**) or in combination with alcian blue (**Figures S1B, E**) which is specific for cartilage, we found that the ossification of the notochord was significantly lower in *lrp5^-/-^* fish at 7dpf and 13dpf compared with siblings. Interestingly, no obvious malformation or mispatterning of the cartilage was observed at these stages (**Figures S1C, F**). Whole-mount alizarin red staining of older juvenile stages (*e.g*. 1.5 mpf, **Figure 2A**), showed a distinct difference in the extent of mineralization in *lrp5* mutants. In planar intramembranous bones, such as the forming calvaria and lateral bones of the skull (opercle, subopercle) alizarin red stain was not uniform and much fainter than in wildtype siblings. (**Figure 2A**). The pattern of mineralization did not show a restricted pattern as would be expected in case of developmental delay, rather was mottled, showing variation across bones in a splotchy pattern (**Figures 2A-D**). We scanned adult *lrp5* mutants at 3 and 6mpf (**Figure 2E**) by X-ray micro-CT to assess changes in BMD in adults. *lrp5^-/-^* fish demonstrated a general lower whole-body BMD (**Figures 2F, G**) and skull BMD (**Figures 2H, I**) at 3 and 6mpf compared to sibling controls. Thus, consistent with findings from other vertebrates and mutant lines of zebrafish, *lrp5* is necessary to establish and maintain BMD.

**Figure 3:**
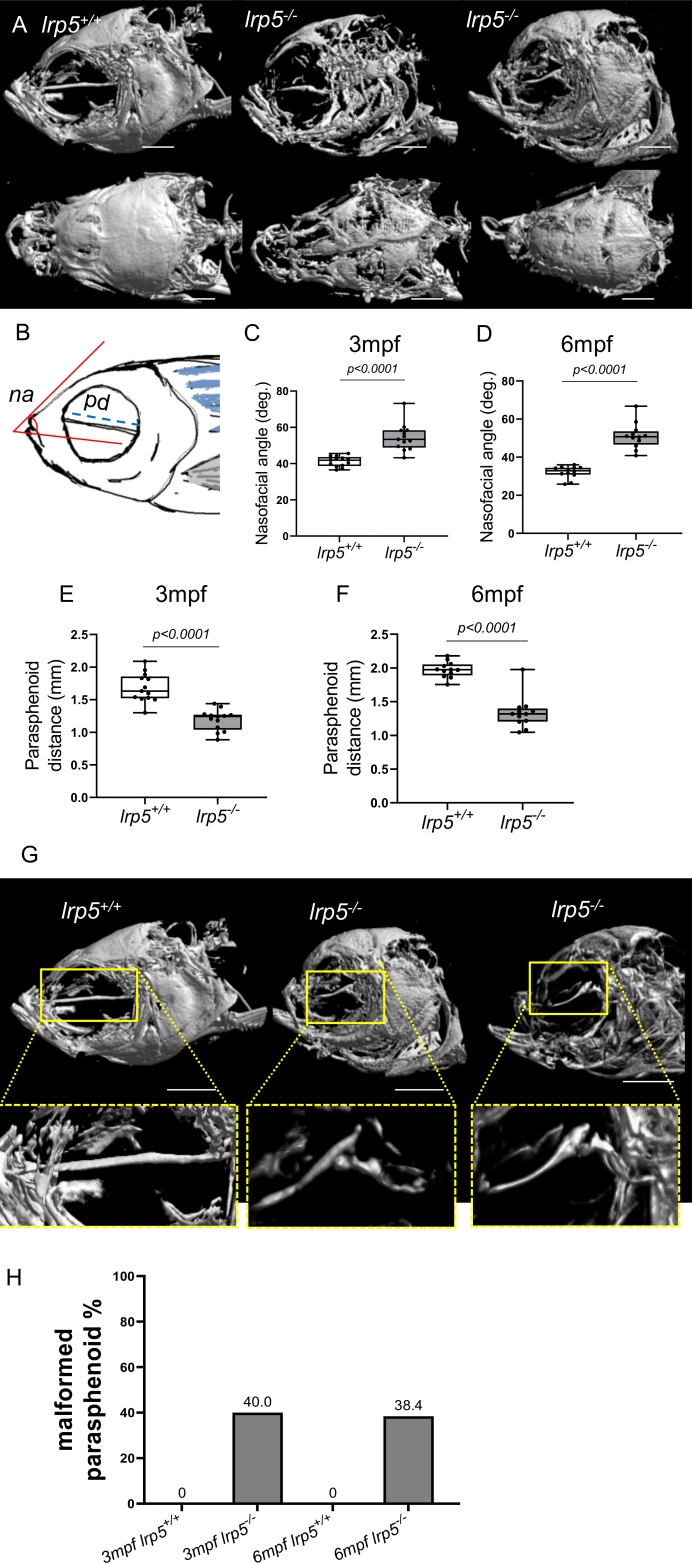
Craniofacial deformities of adult lrp5 mutant. **(A)** Adult *lrp5^-/-^* display major deformities of neuro- and viscera- craniums. **(B)** Schematic representation of morphometrical parameters measured na- nasofacial angle, pd- parasphenoid distance between its edges. **(C, D)** Nasofacial angle at 3mpf and 6mpf is significantly bigger in *lrp5^-/-^* compared to *lrp5^+/+^*, meanwhile parasphenoid distance is significantly shorter in *lrp5^-/-^* adult fish at 3 and 6mpf **(E, F)**. **(G)** Parasphenoid bones of *lrp5^-/-^* are severely deformed and display a similar fracture pattern, outlined in yellow. **(H)** 40% of *lrp5^-/-^* fish displayed malformed parasphenoid at 3mpf and 38.4% at 6mpf within *lrp5^-/-^* group. At 3mpf and 6mpf *lrp5^+/+^* display no indication of parasphenoid deformities.(Scale bar 500µm, 3mpf n =13-15 fish, 6mpf n =12-13 fish, t-test).

As previously noted (39), adult *lrp5^-/-^* fish displayed an abnormal skull shape and body curvatures with variable degree of severity (**Figure 2D**). *lrp5^-/-^* displayed major deformities in the craniofacial skeleton as the cranial skeleton develops asymmetrically with malformations throughout the whole head (**Figure 3A**). To better characterize the deformities in the axial skeleton we segmented the reconstructed micro-CT scans of 3 and 6 mpf fish and performed morphometrical analysis (**Figure 3B**). At both ages, we observed a domed shape cranial vault with anterior protrusion of the frontal bone in *lrp5^-/-^*. Following the same principle as Kague at al. we quantified midface dysmorphology (40) using the nasofacial angle between the maxilla and frontal bones. We measured the nasofacial angle between the parasphenoid, maxilla and frontal bone (**Figure 3B**, outlined in red) and found it was significantly larger in *lrp5* mutants compared to wildtype siblings at 3 (**Figure 3C**) and 6 mpf (**Figure 3D**). Normal frontonasal growth during development is dependent on appropriate extension of the paraphsenoid bone of the chondrocranium. Consistent with the domed shortened skulls of the mutant, we observed that the parasphenoid bone in *lrp5* mutants fish were severely bent, limiting the distance between rostral and caudal extension of the element (**Figure 3B**, blue dashed line; **Figures 3E, F**). Often, the defects were observed as a severe bend of the parasphenoid bone (**Figure 3G**, outlined in yellow) with varied penetrance, 40% of mutants at 3mpf (n=15) while *lrp5^+/+^* sibling fish did not show any abnormalities in the parasphenoid at comparable age.

### lrp5 downstream Wnt-pathway members were not altered in adult *lrp5^-/-^* fish

Lrp5 is a co-receptor of the Wnt-pathway and known to control cell proliferation, migration and participate in expression of osteogenic genes (23, 38, 41). We decided to check the dynamics of Wnt-signaling regulators in response to loss of *lrp5* in the zebrafish. Protein expression levels of β-catenin and phospho-Gsk3β were not altered in *lrp5* mutants compared with *lrp5^+/+^* siblings (**Figure S2A**). Similar expression patterns were observed for β-catenin mRNA (**Figure S2B**). To further assess if the general response of Wnt signaling regulators was altered in the *lrp5* mutant, we checked gene expression response during fin regeneration, when active bone growth is taking place. Higher expression of Wnt-pathway genes, such as *axin2, dkk1a and lef1*, was found in 4 days post amputation (dpa) mutant and wildtype fins compared to intact fins. Interestingly, the expression of Wnt-pathway genes was significantly downregulated in 4dpa regenerate tissue of *lrp5^-/-^* compared to wildtype (**Figures S2C-E**).

### Differential gene expression profiles in *lrp5^-/-^* mutants point to osteoclast deficiencies

**Figure 4:**
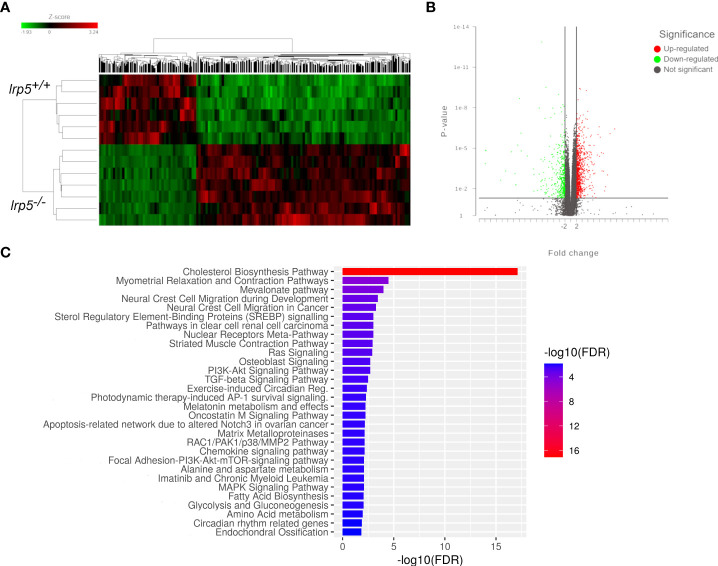
RNA-seq results. **(A)** The overall distribution of differentially expressed genes in the cranial bones between the *lrp5^+/+^* and *lrp5^−/−^* groups. Red and green represent up-regulated and down-regulated transcripts, respectively, in the clustering analysis. The color intensity is directly proportional to the change. **(B)** The volcano plot shows DEGs in the *lrp5^−/−^* cranial bones. The red part indicates up-regulated genes, and the green part indicates down-regulated genes. **(C)** Box plot of pathway analysis for all DEGs.

These data suggest that specific signaling profiles were altered in the *lrp5* mutants during development. To further expand this analysis into bone growth and homeostasis, we assessed the transcriptome of the adult cranium in Lrp5 mutants (n=8) and wildtype siblings (n=6). In total 1044 genes were differentially expressed between *lrp5* mutant and wildtype adult skulls (**Figure 4A**): 725 upregulated and 319 downregulated. Volcano plots were constructed by integrating the p-value and fold change of each transcript to highlight differentially expressed genes (DEGs) between *lrp5* mutant and siblings (**Figure 4B**).

**Figure 5:**
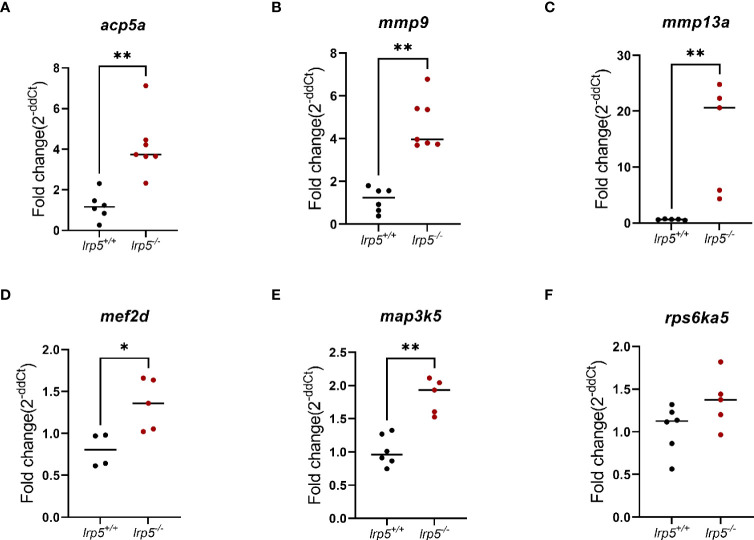
RT-qPCR validation of RNA-Seq data. Genes relevant to osteoclasts activity **(A-C)** and osteoclasts differentiation **(D-F)**. (n=6-7, Mann-Whitney test, dots represent each individual value distributed around mean (line): in black *lrp5^+/+^* control, dark red *lrp5^-/^* ^-^). Mann-Whitney test p-values: *p ≤ 0.05, **p≤ 0.01.

Using pathway analysis of differentially expressed genes, we observed a significant enrichment of genes involved in osteoblast signaling and function and in regulation of osteoclast differentiation such as mevalonate, TGF-beta, p38, MAPK pathways (**Figure 4C**). In parallel, we noted an increase in expression of genes implicated in osteoclast differentiation and activity such as *colony stimulating factor 1 receptor a (csf1ra)*, *acp5a* (encoding TRAP, tartrate resistant acid phosphatase), *tcirg1b* (encoding the a3 isoform of vacuolar H+-ATPase), matrix metalloproteases *mmp9*, *mmp13a*, and *osteoclast stimulation factor 1* (*ostf1*) (**Table 1**). RT-qPCR analysis was used to confirm the expression level of *acp5a, mmp9, mmp13a, mef2d, map3k5*, and *rps6k5* confirming these differential expression results (**Figures 5A-F**).

**Table 1:** Differentially expressed genes related to osteoblasts and osteoclast signaling.

Osteoclast expressed			Osteoblast/osteocyte expressed		
*Gene*	Foldchange	function	*Gene*	Fold change	function
*acp5a*	3.5	Degradation of skeletal phosphoproteins	*bglap*	-2.78	Binds calcium and hydroxyapatite
*mmp9*	2.23	Collagenase, cleaves galectin-3 > suppressor of osteoclastogenesis	*col1a1*	-6.31	Structural component of bone matrix
*fdps*	-3.68	isoprenoid biosynthesis > catalyzes the formation of farnesyl diphosphate	*fgf23*	2.69	Inhibitor of mineralization
*ostf1*	2.09	osteoclast stimulating factor 1	*mmp13a*	10.69	Activates mmp9
*tnfrsf11b*	2.02	Osteoprotegerin acts as decoy receptor for TNFSF11 (RANKL)			

### *lrp5* function is necessary to restrict resorptive activity

**Figure 6:**
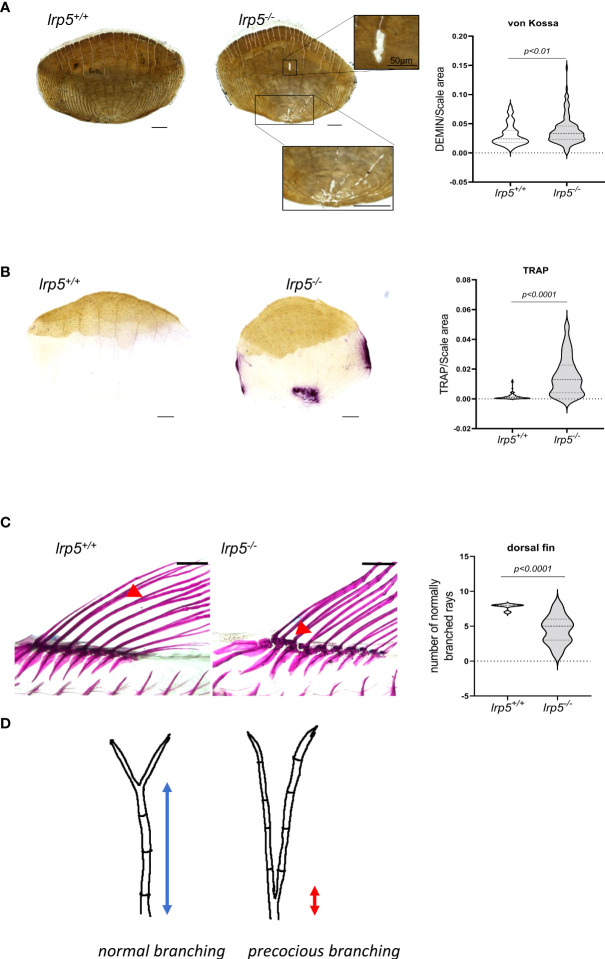
lrp5^-/-^ scales display a larger demineralized area and TRAP positive area. **(A)** von Kossa staining of lrp5^-/-^ scales revealed significantly more demineralized area (t-test, n of scales per group=120-130) than of control siblings scales. **(B)** TRAP staining of *lrp5^-/-^* scales indicates significantly larger TRAP area compared to *lrp5^+/+^* (t-test, n of scales=95-101). Scale bar=100µm. **(C)** Branching of lepidotrichia in *lrp5^+/+^* fins occurs in distal segments (red arrows), meanwhile *lrp5^-/-^* fish display bifurcation in proximal segments of lepidotrichia (C, red arrows). The number of normally branched fin rays is significantly lower in *lrp5^-/-^* compared to *lrp5^+/+^* siblings. **(D)** Schematic representation of excessive branching in *lrp5^-/-^*. Results in A, B and C are expressed as mean SD.

The enrichment of DEGs with regulatory pathways of osteoclast function was unexpected. As such, we decided to check the osteoclast activity in the adult skeleton of *lrp5* mutants compared to wildtype siblings. As a proxy for general resorptive activity more broadly, we analyzed remodeling within zebrafish elasmoid scales. Zebrafish elasmoid scales are translucent mineralized structures of the dermal skeleton with osteoblasts and osteoclasts present (42). Over the last decade zebrafish scales have been used to exhibit the mechanism of bone remodeling (34, 43, 44). Using Von Kossa staining, we observed a distinctive demineralized area at the base of scales in 77% of *lrp5* mutant scales (n=134), compared to just 22% in wildtype siblings (n= 118) (**Figure 6A**). We quantified demineralization, normalizing it to the size of scales, and found that the demineralized area was significantly larger in mutant scales (*p=0.004386, t-test*) (**Figure 6A**). We further decided to check osteoclast activity using TRAP staining across an area supporting increased osteoclast activity. In line with the observed increase in bone resorption in the mutant, we observed a significant increase of TRAP staining in *lrp5^-/-^* scales suggesting higher osteoclast activity in the absence of Lrp5 signaling (p<0.0001, t-test) (**Figure 6B**).

### The dermal rays of fish fins show branching of the lepidotrichial skeletal elements

Recent work has shown that lepidotrichia branching process is sensitive to a balance between osteoblast-mediated bone growth and osteolytic tubules expressing *ctsk* and *trap* (45). The extent of branching is sensitive to drugs modifying osteoclast behavior and acts as a barometer of osteoclast activity in the development of the zebrafish skeleton. Consistent with this new finding, whole-mount alizarin red staining of adult *lrp5* mutant fish revealed abnormal branching of lepidotrichia in median fins (**Figure 6C**). In wildtype fins, the bifurcation or splitting of lepidotrichia demonstrates an organized pattern, occurring in a distal segment of the ray (**Figure 6C**, red arrow). Meanwhile in fins of *lrp5* mutants various fin rays lack ‘stitching’ of the hemirays (45) causing ray separation in the proximal segment of the lepidotrichia (**Figure 6C**). We quantified the number of normally branched fin rays in dorsal fins of mutants and compared it to wildtype siblings. We found that dorsal fins of *lrp5* mutant fish display significantly less normal branched fin rays compared to wildtype siblings (**Figure 6C**, p<0.0001, t-test). The precocious branching is consistent with the increased bone reworking and TRAP staining in scales and suggests activation of osteoclasts in *lrp5*-deficient mutant fish.

## Discussion

In humans, recessive loss-of-function mutations in *LRP5*, a co-receptor in the Wnt signaling pathway, cause osteoporosis-pseudoglioma syndrome. Several GWAS studies identified *LRP5* as a major risk locus for osteoporosis-related phenotypes in the general population (10–12). Prior work detailed similar action of Lrp5 in the zebrafish regulating bone mineral density (39). However, the causes of these broad and systemic phenotypes have not been identified. To further address how Lrp5 regulates skeletal homeostasis, we created a zebrafish *lrp5* loss of function mutant, and looked at changes in timing of ossification and changes in transcriptional regulation that may provide insight into its action in patterning and remodeling of bone. Our findings point to a specific role of Lrp5 in suppressing osteoclast function during the development of new bone and in adult skeletal elements. Alteration of Lrp5 activity may also contribute to the loss of skeletal integrity.

We found that Lrp5 was essential for early viability as only 3.7% of homozygotes survived and reached sexual maturity. The low survival rates in the mutants may be due to alterations in cranial neural crest specification (38) making *lrp5^-/-^* more vulnerable. However, we did not see any apparent cartilage patterning defects at these early stages. By avoiding constant water current and providing delicate feeding during the first 35 days, we were able to increase survival rates of *lrp5* mutant fish. The mutants that survived early development were then able to thrive into adulthood and reproduce.

Late skeletal roles of Lrp5 were apparent in the growth and differentiation of skeletal elements. In *lrp5* mutant juveniles, we observed an altered mineralization pattern of the craniofacial skeleton. At adult stages, *lrp5^-/-^* fish demonstrated a decrease in whole-body BMD and a more drastic decrease in the BMD level of the head skeleton. These findings are consistent with a conserved role of Lrp5 in regulating mineralization and mineral density (46). The demonstration of progressive deficiencies in mineralization during development in the *lrp5* mutant provides an interesting experimental tool to assess progressive action of Lrp5 in regulating this trait.

Recently it was reported that *lrp5* mutant fish display craniofacial malformations in the adult, consistent with defects in skeletal integrity (39). However, we observed substantially more severe malformations in our *lrp5^-/-^* fish suggesting a role of Lrp5 in developmental patterning. It is plausible to assume that the higher penetrance of the craniofacial phenotype may be associated with the position of the induced mutation near the beginning of the protein, which results in appearance of a stop codon at the first LY domain, limiting the retention of all functional domains. In contrast, the previously reported zebrafish mutants carry the mutation in exon 5 that creates a stop codon at amino acid position 284, meanwhile zebrafish mutants we report here carry the mutation in exon 2 with stop codon at 54 aa. Although, early embryos look morphologically normal, adults have penetrant dysmorphologies of a domed cranial vault, anterior protrusion of the frontal bone and hypoplasia of facial bones. The nasofacial angle was significantly obtuse in crania of adult *lrp5* mutants consistent with decreased frontonasal growth and bossing. A key structural aspect of frontonasal growth is the formation and extension of the parasphenoid of the chondrocranium of the skull. In *lrp5* mutants, we observed severely bent and kinked parasphenoid bones. In similar vein, we find that the *lrp5* mutants have varied levels of fractures in craniofacial bones as seen in the dentary and in the parasphenoid, suggesting skeletal fragility in the mutants.

Insight into the mechanistic regulation of Lrp5 signaling in late development was brought out through our analysis of transcriptional regulation in resting bones of the skull. *lrp5* mutants exhibited lower expression of osteoblastic markers such as *bglap, fgf23* and *col1a1*. Extracellular matrix proteins such as *osteocalcin* (*bglap*) are generally expressed by mature and resting osteoblasts and by hypertrophic chondrocytes. Notably, in contrast, we observed upregulation of genes associated with osteoclast activity, such as *acp5a, tcirg* and *mmp9.*The *acp5a* gene in zebrafish encodes TRAP (tartrate-resistant acid phosphatase), which is secreted by macrophages, dendritic cells and most importantly by bone-resorbing osteoclasts (47, 48). TRAP is one of the key enzymes of the osteoclast resorption process and considered as a classical marker to measure osteoclast activity and number. No less importantly we observed the upregulation of the osteoclast-specific subunit of the v-ATPase *tcirg1*, which is essential for the acidification of the resorption lacuna (49, 50) and *mmp9* expression, an osteoclast-secreted protein known to degrade the bone collagenous matrix (51, 52). We show that pathways responsible for osteoclastogenesis are differentially expressed in *lrp5* mutants such as mevalonate, p38, MAPK and TGF-beta signaling pathways. One of the important roles of the mevalonate pathway is inhibition of osteoclast development *via* farnesyl diphosphate synthase (*fdps*) (53) and we found *fdps* 3.68-fold downregulated in cranial bones of *lrp5* mutant fish (**Table 1**). In addition, we found a significant increase in *csf1ra.* Csf1ra is the receptor for macrophage colony-stimulating factor 1 (m-csf) which is essential for macrophage differentiation to osteoclast (54). Together these data suggest that *lrp5* has an essential role in suppressing osteoclast regulation during bone homeostasis. To functionally assess this, we employed zebrafish scales as a model to observe osteoclast resorptive activity. The resorption area in *lrp5^-/-^* scales is significantly larger in mutants consistent with increased activity. This is further corroborated by detection of higher TRAP signal corresponding to increased osteoclast activity (**Figure 6B**). Osteoclasts are active in shaping developing and remodeled bone, as well as in repair of bone after damage. One emerging anatomical readout of osteoclast activity is the formation and the position of branch sites in the dermal rays of the zebrafish fin. Normally bifurcation of the fin rays occurs in the distal segments of lepidotrichia (**Figure 6D**). This process is mediated by bone resorption by osteolytic cells that facilitate the shaping of the lepidotrichia (55). Consistent with this morphological readout of osteoclast activity, we found that the number of normally branched fin rays was lower in *lrp5* mutants and deviated from the otherwise determinative pattern of branching within the fin.

The canonical Wnt-pathway is known to inhibit osteoclastogenesis *via* activated Wnt/β-catenin signals (56). Given our observed role of Lrp5 in suppressing osteoclast function we assessed the effect on Wnt downstream targets that may underlie these observations. In adult skeletal structures, we found that the protein expression levels of β-catenin and phospho-Gsk3β were similar in mutants and wildtype siblings. We further tested expression of selected Wnt-pathway genes during bone regeneration during phases of active bone growth (57). In contrast to the resting adult skeleton, during formation of new bone the expression of selected Wnt-pathway genes was significantly downregulated in *lrp5* mutant caudal fin regenerates compared to wildtype regeneration controls. In particular, the expression of *lef1* was significantly downregulated. Thus, we suggest that the canonical Wnt-pathway is attenuated in the developing skeleton of *lrp5* mutants suggesting potential mechanisms for regulation of osteoclast differentiation during development. This does not explain the difference in osteoclast activity in resting bones, however.

While we do show an increase in resorptive activity through von Kossa and TRAP staining of scales and dermal ray branching, it is unclear if this increase is caused by an elevated osteoclast number or increased cell’ activity. Investigation of osteoclast number during development of *lrp5* mutants from early to adult stages will be important to discern between a role of Lrp5 in osteoclastogenesis or in regulation of osteoclast activity.

In summary, our analysis demonstrates that *lrp5* deficiency results in delayed mineralization throughout the zebrafish skeleton during development consistent with loss of BMD at adult stages. This mineral deficiency is especially visible in the formation of the cranial skeleton, and results in the deformation of the viscerocranium and frontonasal shortening. We show that in-depth transcriptome analysis of cranial bones highlights differential regulation of genes associated with osteoclastogenesis in *lrp5* mutant tissues. We were able to confirm specific osteoclast regulation by demonstrating the increase of osteoclast activity and high resorption rate in *lrp5* mutant skeletal tissue that leads to a specific developmental readout of differential branching of dermal rays of the fin. Our study revealed unexpected insights into the role of Lrp5 in bone homeostasis through moderation of osteoclast function. Altogether our model permits characterization of the craniofacial skeleton and supports a new hypothesis for Lrp5 in the regulation of skeletal form and function. Thus, we propose *lrp5 *knockout as an experimentally tractable model to examine post-embryonic bone development and the functional role of lrp5 signaling in osteoclast metabolism.

## Data availability statement

The datasets presented in this study can be found in online repositories. The names of the repository/repositories and accession number(s) can be found in the article/**Supplementary Material**.

## Ethics statement

The animal study was reviewed and approved by Institutional Animal Care and Use Committees of Israel.

## Author contributions

IK: conceptualization, investigation, data curation, formal analysis, methodology, validation, visualization, writing-original draft, and writing-review and editing. CS-C: conceptualization, data curation, methodology, resources, visualization, and writing-review and editing. RH: formal analysis and investigation. KH: data analysis, methodology, and writing-review and editing. KW: data analysis and methodology. MH: conceptualization, supervision, and writing-review and editing. DK: conceptualization, data curation, funding acquisition, project administration, supervision, and writing-review and editing. All authors contributed to the article and approved the submitted version.

## Funding

The study was supported by a grant No. 2017204 from US-Israel BSF and grant No. 1121/19 from Israel Science Foundation.

## Acknowledgments

The authors express their gratitude to Inbar Ben-Zvi and Malka Kitainer, PhD for expert technical support. We also thank Dalia David for helpful discussions.

## Conflict of interest

The authors declare that the research was conducted in the absence of any commercial or financial relationships that could be construed as a potential conflict of interest.

The handling editor EK declared a shared committee Consensus statement on bone-microCT measurement standardization with the author DK at the time of review.

## Publisher’s note

All claims expressed in this article are solely those of the authors and do not necessarily represent those of their affiliated organizations, or those of the publisher, the editors and the reviewers. Any product that may be evaluated in this article, or claim that may be made by its manufacturer, is not guaranteed or endorsed by the publisher.

## Supplementary material

Supplementary Figure 1 Lrp5 mutants display delayed mineralization at early developmental stages. Alizarin red staining together with alcian blue double staining shows significantly lower mineralization level of the notochord (yellow, dashed) and at 7 (A-C) and 13 (D-F) days post fertilization (dpf) compared with lrp5^+/+^ siblings (7dpf n=37-44, 13dpf n=24-25, Mann-Whitney test, measured area outlined in yellow). Scale bar=100µm.

Supplementary Figure 2 Wnt-signaling pathway genes activity in lrp5^-/-^ homozygous fish. (A) Wnt-pathway proteins in *lrp5^-/-^* fish, compared to *lrp5^+/+^* siblings. (B) RT-qPCR for expression of β-catenin gene in *lrp5* ^-/-^ caudal fins compared to *lrp5^+/+^* siblings. (C-E) mRNA level of Wnt-pathway genes is elevated in zebrafish regenerated caudal fins, compared to intact fins (n=5 fish in each group, Mann-Whitney test).
