# Supplementary figures and images for: Zebrafish mutants reveal unexpected role of Lrp5 in osteoclast regulation
Source: Front Endocrinol (Lausanne). 2022 Sep 2;13:985304. doi: 10.3389/fendo.2022.985304 (PMC9478031; doi:10.3389/fendo.2022.985304)

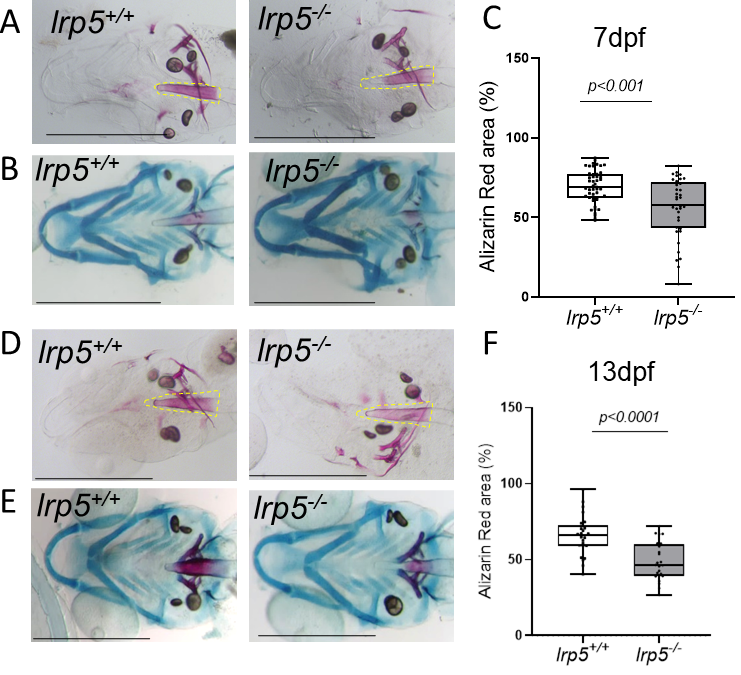

Supplement: Supplementary Figure 1 — Lrp5 mutants display delayed mineralization at early developmental stages. Alizarin red staining together with alcian blue double staining shows significantly lower mineralization level of the notochord (yellow, dashed) and at 7 (A-C) and 13 (D-F) days post fertilization (dpf) compared with lrp5+/+ siblings (7dpf n=37-44, 13dpf n=24-25, Mann-Whitney test, measured area outlined in yellow). Scale bar=100µm. [file Image_1.tif]

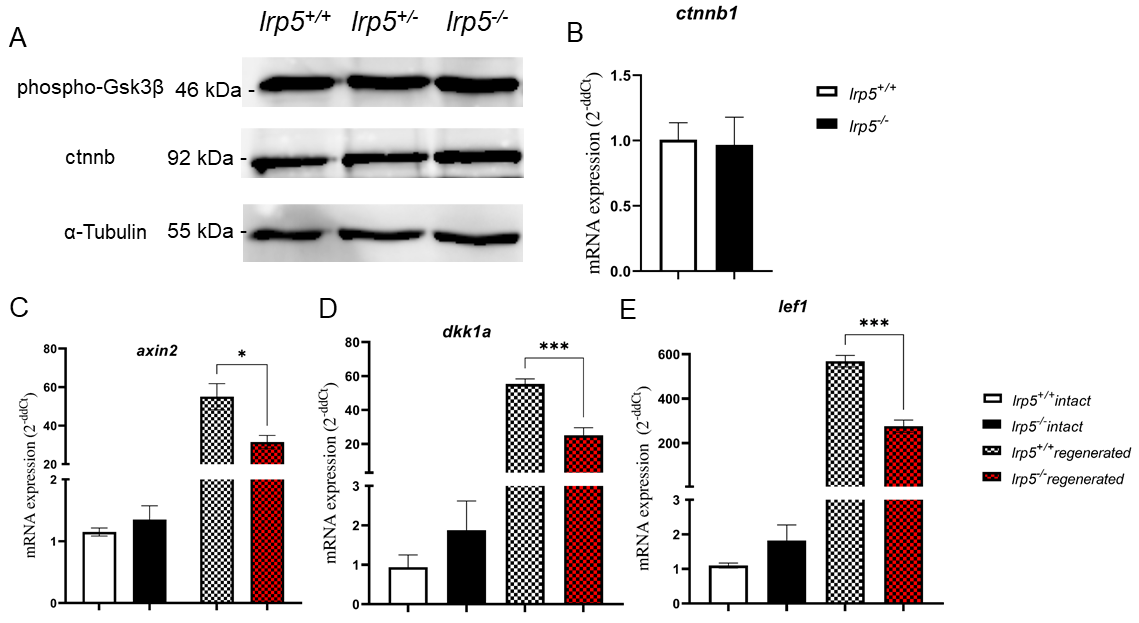

Supplement: Supplementary Figure 2 — Wnt-signaling pathway genes activity in lrp5-/- homozygous fish. (A) Wnt-pathway proteins in lrp5-/- fish, compared to lrp5+/+ siblings. (B) RT-qPCR for expression of β-catenin gene in lrp5 -/- caudal fins compared to lrp5+/+ siblings. (C-E) mRNA level of Wnt-pathway genes is elevated in zebrafish regenerated caudal fins, compared to intact fins (n=5 fish in each group, Mann-Whitney test). [file Image_2.tif]
