# Supplementary material for: Zebrafish mutants reveal unexpected role of Lrp5 in osteoclast regulation
Source: Front Endocrinol (Lausanne). 2022 Sep 2;13:985304. doi: 10.3389/fendo.2022.985304 (PMC9478031; doi:10.3389/fendo.2022.985304)
Supplement: Supplementary file 3 [file DataSheet_1.pdf]

Supplemental Table 1. Primers used for genotyping

Gene name	Forward	Reverse
lrp5	GCTGGTTGTTTGTTCAAATGC	GAAATCATCGAAGCCACAAAA

Supplemental Table 2. Primers used for qPCR

Gene name	Forward	Reverse
18s	TCGCTAGTTGGCATCGTTTATG	CGGAGGTTCGAAGACGATCA
acp5a	GCAGAAGCTGCTGGTATGGC	TAGCAAAGGACCCCAGGGA
map3k5	TGTCAGAAGAATATGACCTGCG	CCTTACTCTGAGGCGTCACC
mef2d	GGAGTTGGTGAGTGAGAATGC	CCACTGGTGTGGTAAGAGGC
mmp13a	CAGCCTTCCAGTACAGAGGTTT	ATTGTTGTTCAGGACGCGGA
mmp9	CTTCAAGGACGGGCGCTACT	GGTGGTCCTCAAAGGCAGAG
rps6ka5	CTTTGCGTTAGTGTCGTGCC	TGACCCGTTAGATTGGCGTT
